# Supplementary material for: β4GALT1 controls β1 integrin function to govern thrombopoiesis and hematopoietic stem cell homeostasis
Source: Nat Commun. 2020 Jan 17;11:356. doi: 10.1038/s41467-019-14178-y (PMC6968998; doi:10.1038/s41467-019-14178-y)
Supplement: Supplementary file 1 — Supplementary Information [file 41467_2019_14178_MOESM1_ESM.pdf]

β4GALT1 controls β1 integrin function to govern thrombopoiesis and hematopoietic stem cell homeostasis.

Giannini S., *et al*.

Supplementary Table 1: Subset of hematopoietic stem and progenitor cells and phenotypic definition

Cell population	Definition
LSK	Lin^-, $cKit^+$, $Sca1^+$
LT-HSC	Lin^-, $cKit^+$, $Sca1^+$, $CD48^-$, $CD150^+$
ST-HSC	Lin^-, $cKit^+$, $Sca1^+$, $CD48^-$, $CD150^-$
MPP	Lin^-, $cKit^+$, $Sca1^+$, $CD48^+$
PreMegE	$cKit^+$, $Sca1^-$, $CD41^-$, FcgRI/IIlo, $CD105^+$, $CD150^-$
MKP	Lin^-, $cKit^+$, $Sca1^-$, $CD41^+$, $CD150^+$
CMP	Lin^-, $cKit^+$, $Sca1^-$, $CD34^+$, $FcgRI/II^-$
GMP	Lin^-, $cKit^+$, $Sca1^-$, $CD34^+$, $FcgRI/II^+$

Supplementary Table 2: List and sequence of primers

Primer	Sequence
B4galt1	
B4galt1$^{+/+}$ F	CACCCCTTAGACTCCGCC
B4galt1$^{+/+}$ R	AAACCTCATCTTCCCGCCG
B4galt1$^{-/-}$ F	CCTGAATGAACTGCAGGACG
B4galt1$^{-/-}$ R	AGTGACAACGTCGAGCACAG
PF4-Cre	
Control F	GGCAAAGGTGGAAATGAAGA
Control R	CTCAGACCACACAGGGAATG
PF4-Cre F	TGGGAGAATGCTGATCCACA
PF4-Cre R	TCCATCTCTCCACCAGCTTG
Itgb1	
Itgb1 F	CCACAACTTTCCCAGTTAGCTCTC
Itgb1 R	CCACAACTTTCCCAGTTAGCTCTC
B4galt1 mRNA	
B4galt1$^{+/+}$ F	GCAACTCGACTATGGCATCTACG
B4galt1$^{+/+}$ R	CGGAATGAGGTCCACATCATG
GAPDH mRNA	
GAPDH F	CGACTTCAACAGCACTCCCACTCTTCC
GAPDH R	GGGTGGTCCAGGGTTTCTTACTCCTT

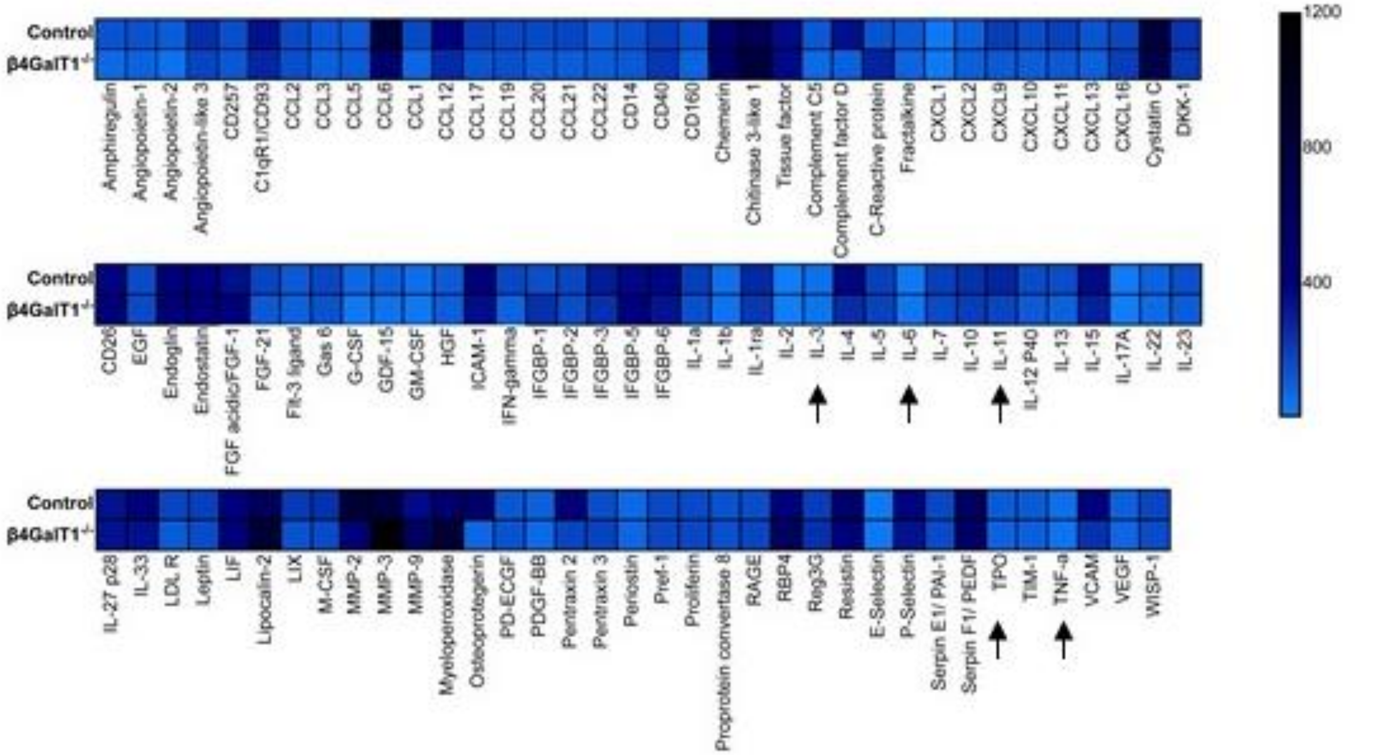

Supplementary Figure 1: Cytokine array of control and *B4galt1*$^{-/-}$ BM supernatants.

Heat map representing cytokine and chemokine levels measured in the BM supernatant of control and *B4galt1*$^{-/-}$ mice. n ≥ 5 in each group.

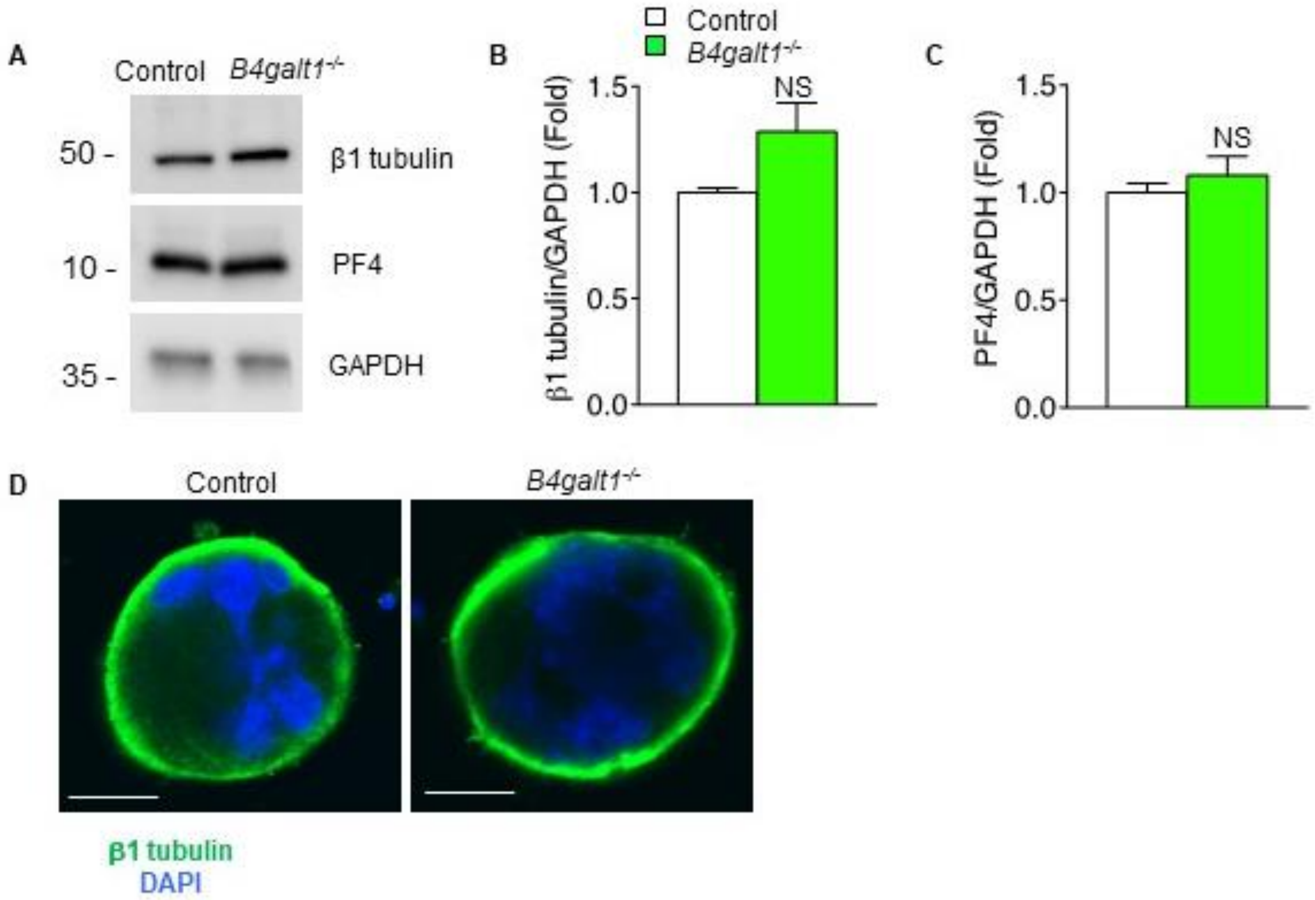

Supplementary Figure 2: β4GalT1$^{-/-}$ MKs express normal levels of lineage specific markers.

(A) *B4galt1*$^{-/-}$ and control MK lysates were subjected to SDS-PAGE and immunoblotting using anti β1 tubulin, PF4 and GAPDH mABs. (B-C) Densitometric quantification of control (white) and *B4galt1*$^{-/-}$ (green) MK immunoblots probed with anti-β1 tubulin and anti-PF4 mAb. Data are expressed as mean ± SEM. n = 3 in each group. Groups were compared using an unpaired Student's t-test. NS= not significant. (D) Immunofluorescence of control and *B4galt1*$^{-/-}$ MKs stained with anti-β1 tubulin antibody (green) and DAPI (blue); scale bar: 10 μm. The image is representative of 5 different megakaryocytes from 2 different mice/genotype.

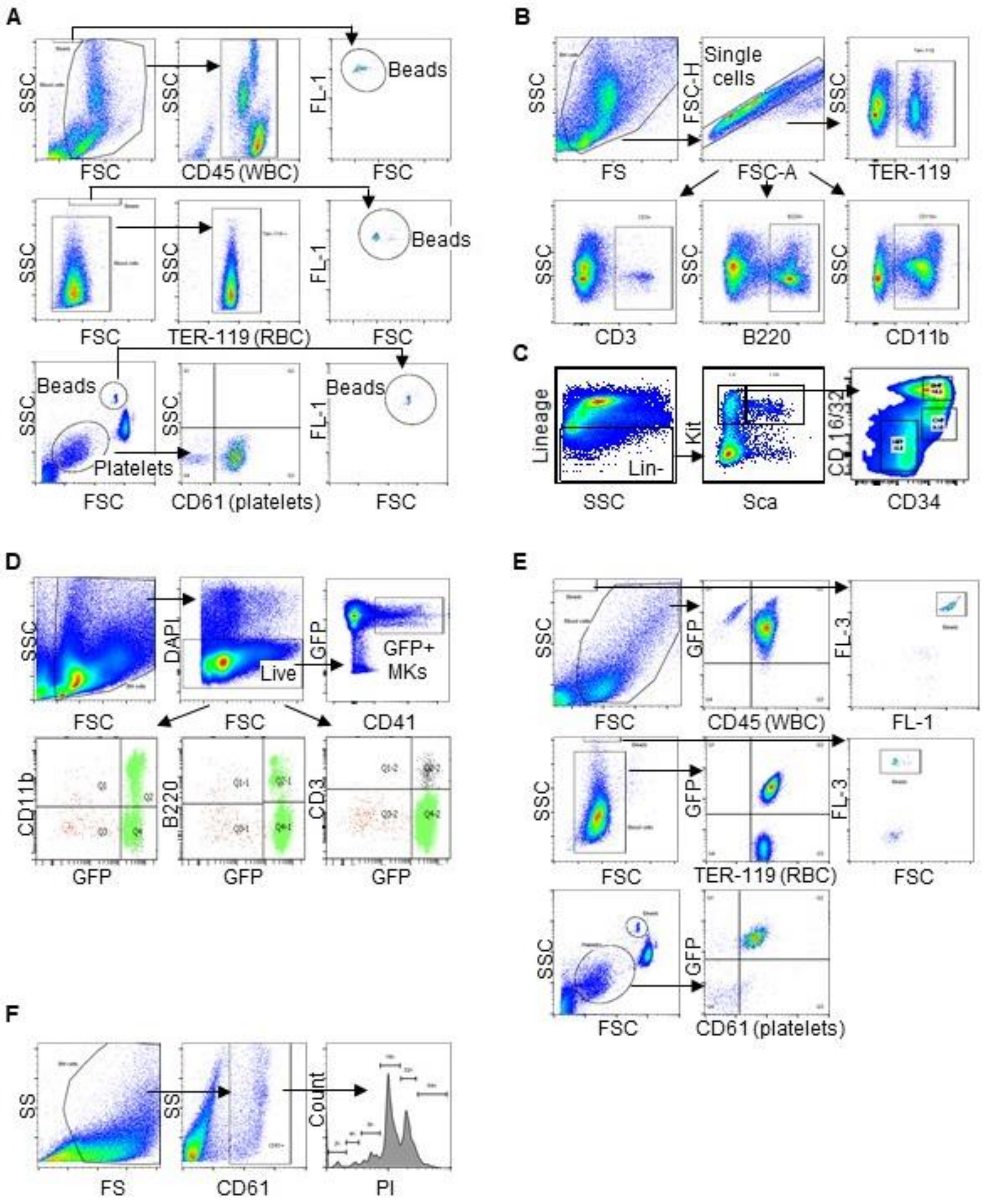

Supplementary Figure 3: Gating strategies.

(A) Gating strategies for blood cell count in Figure 1. WBCs, RBCs and platelets were identified based on light scatters and positivity for specific markers. Cell count was calculated based upon the concentration of reference beads. (B-C) Gating strategy used to analyze BM cells in Figure 2. (B) BM cells were stained with lineage specific markers TER-119, CD3, B220. (C) Gating strategy to analyze hematopoietic progenitors in Figure 2C. (D-E) Gating strategy utilized to quantify donor derived cell populations following HSC transplantation into WT recipients as shown in Figure 3. (D) BM cells were stained with CD41 to identify GFP+ MKs. (E) Blood cells were stained for lineage specific markers to count total and GFP+ WBCs, RBCs and platelets. (F) Gating strategy used to quantify MK ploidy in Figure 4. Following RNA digestion, BM cells were stained with anti-CD41 to identify MKs and with PI to determine their DNA content.

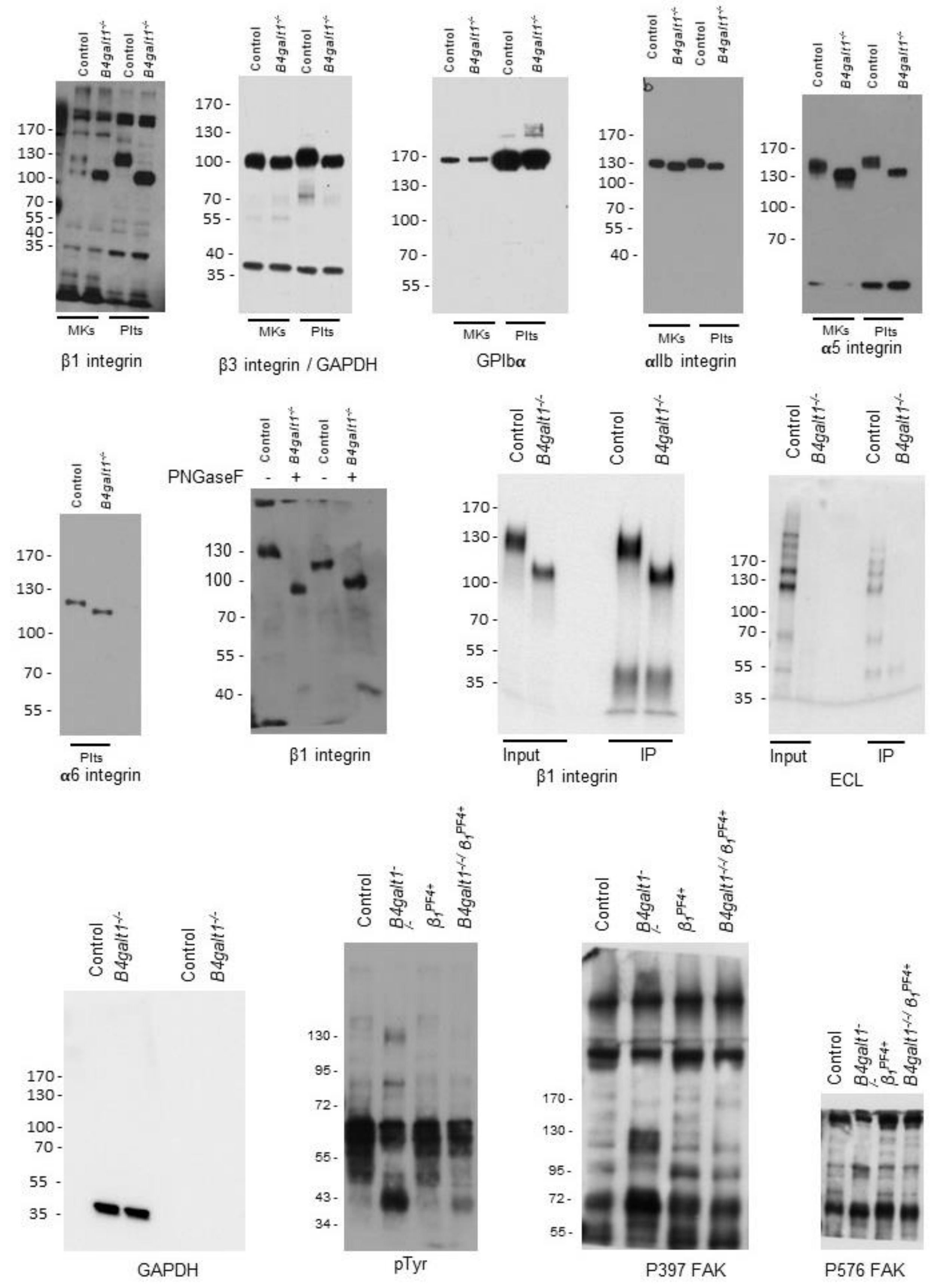

Supplementary Figure 4: Full blots shown in Figure 5.

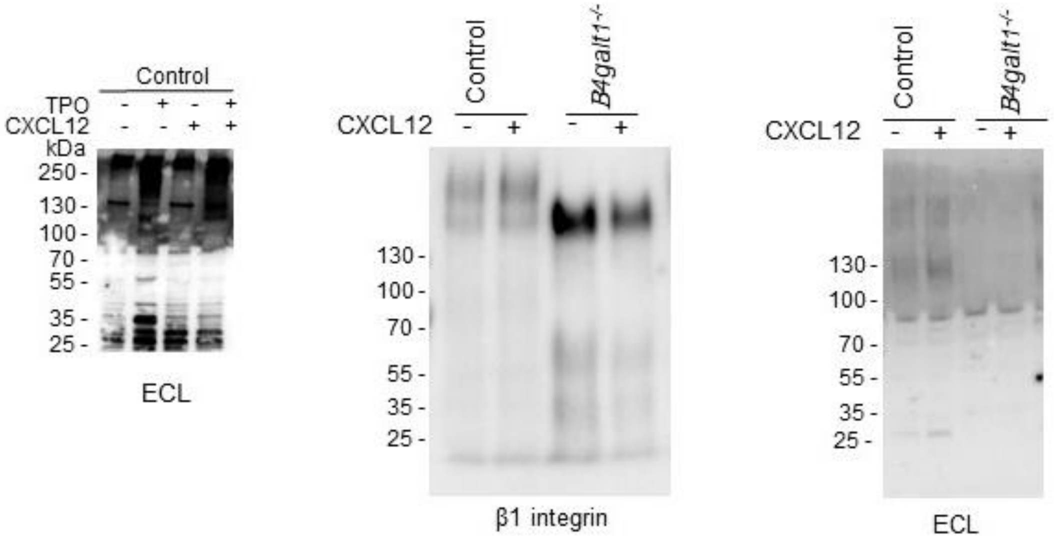

Supplementary Figure 5: Full blots shown in Figure 7.

Supplementary Methods:

Illumina RNAseq analysis

To compare the expression level of *B4galt1* and *B4galt2* across murine blood cell types we analyzed publically available Illumina RNAseq datasets. Fastq files containing sequence reads (suppletmentary table 3) were obtained for MKs[1] (Encode experiment: ENCSR025JRA), platelets[2] (NCBI Bioproject: PRJNA413262), LSK cells[3] (NCBI Bioproject: PRJNA208010), dendritic cells[1] (Encode experiment: ENCSR945SYV), and regulatory T cells[1] (Encode experiment: ENCSR486LMB). All reads were aligned to the GRCm38 transcriptome obtained from Ensembl[4] using Kallisto v0.43.1[5] and read counts were summarized to the gene level using the R v3.4.3[6] and tximport v1.6.0[7]. All libraries were normalized using DESeq2 v1.18.1[8] and FPKM values calculated from these normalized libraries were used in Figure 5E. R Core Team (2017). R: A language and environment for statistical computing. R Foundation for Statistical Computing, Vienna, Austria. URL http://www.R-project.org/.

Supplementary References:

1. The ENCODE Project Consortium. An Integrated Encyclopedia of DNA Elements in the Human Genome. *Nature* **489**, 57-74 (2012).
2. Rowley JW et al. Genome-wide RNA-seq Analysis of Human and Mouse Platelet Transcriptomes. *Blood* **118**, e101-111 (2011).
3. Sun D et al. Epigenomic Profiling of Young and Aged HSCs Reveals Concerted Changes During Aging That Reinforce Self-Renewal. *Cell Stem Cell* **14**, 673-88 (2014).
4. Sun D et al. Epigenomic Profiling of Young and Aged HSCs Reveals Concerted Changes During Aging That Reinforce Self-Renewal. *Cell Stem Cell* **14**, 673-88 (2014).
5. Zerbino DR et al., Ensembl 2018. *Nucleic Acids Res* **46**, D754-761 (2018).
6. Bray NL et al., Near-optimal Probabilistic RNA-seq Quantification. *Nat. Biotechnol* **34**, 525-7 (2016).
7. Soneson C et al., Differential Analyses for RNA-seq: Transcript-Level Estimates Improve Gene-Level Inferences. *F1000Res* **4**, 1521 (2015).
8. Love MI et al Moderated Estimation of Fold Change and Dispersion for RNA-seq Data With DESeq2. *Genome Biol* **15**, 550 (2014).
