# Supplementary material for: β4GALT1 controls β1 integrin function to govern thrombopoiesis and hematopoietic stem cell homeostasis
Source: Nat Commun. 2020 Jan 17;11:356. doi: 10.1038/s41467-019-14178-y (PMC6968998; doi:10.1038/s41467-019-14178-y)
Supplement: Supplementary file 3 — Reporting Summary [file 41467_2019_14178_MOESM3_ESM.pdf]

nature research

Corresponding author(s): Karin Hoffmeister

Last updated by author(s): Nov 21, 2019

Reporting Summary

Nature Research wishes to improve the reproducibility of the work that we publish. This form provides structure for consistency and transparency in reporting. For further information on Nature Research policies, seeAuthors & Referees and theEditorial Policy Checklist .

Statistics

For all statistical analyses, confirm that the following items are present in the figure legend, table legend, main text, or Methods section.

n/a	Confirmed	
☐	☒	The exact sample size (*n*) for each experimental group/condition, given as a discrete number and unit of measurement
☐	☒	A statement on whether measurements were taken from distinct samples or whether the same sample was measured repeatedly
☐	☒	The statistical test(s) used AND whether they are one- or two-sided<br>*Only common tests should be described solely by name; describe more complex techniques in the Methods section.*
☒	☐	A description of all covariates tested
☐	☒	A description of any assumptions or corrections, such as tests of normality and adjustment for multiple comparisons
☐	☒	A full description of the statistical parameters including central tendency (e.g. means) or other basic estimates (e.g. regression coefficient) AND variation (e.g. standard deviation) or associated estimates of uncertainty (e.g. confidence intervals)
☐	☒	For null hypothesis testing, the test statistic (e.g. *F*, *t*, *r*) with confidence intervals, effect sizes, degrees of freedom and *P* value noted<br>*Give P values as exact values whenever suitable.*
☒	☐	For Bayesian analysis, information on the choice of priors and Markov chain Monte Carlo settings
☒	☐	For hierarchical and complex designs, identification of the appropriate level for tests and full reporting of outcomes
☐	☒	Estimates of effect sizes (e.g. Cohen's *d*, Pearson's *r*), indicating how they were calculated

Our web collection on statistics for biologists contains articles on many of the points above.

Software and code

Policy information about availability of computer code

Data collection	To compare the expression level of B4GalT1 and B4GalT2 across murine blood cell types we analyzed publically available Illumina RNAseq datasets. Fastq files containing sequence reads were obtained for MKs (PMID: 22955616; Encode experiment: ENCSR025JRA), platelets (PMID: 21596849; NCBI Bioproject: PRJNA413262), LSK cells (PMID: 24792119; NCBI Bioproject: PRJNA208010), dendritic cells (PMID: 22955616; Encode experiment: ENCSR945SYV), and regulatory T cells (PMID: 22955616; Encode experiment: ENCSR486LMB). All reads were aligned to the GRCm38 transcriptome obtained from Ensembl (PMID: 29155950) using Kallisto v0.43.1 (PMID: 27043002) and read counts were summarized to the gene level using the R v3.4.3 (R Core Team, 2017) and tximport v1.6.0 (PMID: 26925227). All libraries were normalized using DESeq2 v1.18.1 (PMID: 25516281) and FPKM values calculated from these normalized libraries were used in Figure 5E. R Core Team (2017). R: A language and environment for statistical computing. R Foundation for Statistical Computing, Vienna, Austria. URL http://www.R-project.org/.
Data analysis	GraphPad Prism 5<br>Image J

For manuscripts utilizing custom algorithms or software that are central to the research but not yet described in published literature, software must be made available to editors/reviewers. We strongly encourage code deposition in a community repository (e.g. GitHub). See the Nature Research guidelines for submitting code & software for further information.

Data

Policy information about availability of data

All manuscripts must include a data availability statement. This statement should provide the following information, where applicable:

- Accession codes, unique identifiers, or web links for publicly available datasets
- A list of figures that have associated raw data
- A description of any restrictions on data availability

Complied

Field-specific reporting

Please select the one below that is the best fit for your research. If you are not sure, read the appropriate sections before making your selection.

☒ Life sciences ☐ Behavioural & social sciences ☐ Ecological, evolutionary & environmental sciences

For a reference copy of the document with all sections, see nature.com/documents/nr-reporting-summary-flat.pdf

Life sciences study design

All studies must disclose on these points even when the disclosure is negative.

Sample size	In the absence of preliminary results to estimate the effect size and variability of our measure, we have performed several sample size calculations under the assumption that the magnitude of the effect size would be important (assumption which later proved to be correct). Based on our assumptions including distributional assumptions (normal distribution of the mean effect size), we calculated that a sample size of > 3 animals / group would be appropriate for our experiment. Ref: Chow SC, Shao J and Wang H. Sample size calculations in clinical research. 2nd ed. Chapman & Hall/CRC. Boca Raton 2008.
Data exclusions	No data were excluded from the analysis
Replication	Experimental procedures are SOP. Experiments were reproduced by at least by 2 independent investigators.
Randomization	Mice were allocated into experimental groups that were comparable for gender and age
Blinding	Investigators were not blinded to group allocation

Reporting for specific materials, systems and methods

We require information from authors about some types of materials, experimental systems and methods used in many studies. Here, indicate whether each material, system or method listed is relevant to your study. If you are not sure if a list item applies to your research, read the appropriate section before selecting a response.

Materials & experimental systems

n/a	Involved in the study	
☐	☒	Antibodies
☒	☐	Eukaryotic cell lines
☒	☐	Palaeontology
☐	☒	Animals and other organisms
☒	☐	Human research participants
☒	☐	Clinical data

Methods

n/a	Involved in the study	
☒	☐	ChIP-seq
☐	☒	Flow cytometry
☒	☐	MRI-based neuroimaging

Antibodies

Antibodies used

GPIbalpha, Emfret analytics, clone Xiao G5, Cat # M-040
GPIbalpha, Emfret analytics, clone XiaoG7, Cat # M-042
Laminin, Sigma aldrich, polyclonal, Cat# L9393
beata1 integrin, Abcam, polyclonal, Cat# ab183666
beata1 integrin, becton Dickinson, clone Ha2/5, Cat# 555005
Activated beata1 integrin, Becton Dickinson, clone 9EG7, Cat# 553715
CD41, Becton Dickinson, clone MWReg30, Cat# 553847
Ter119, Ebioscience, clone TER-119, Cat. # 48-5921-82
Ly-6G (Gr-1) Ebioscience, clone RB6-8C5, Cat. # 48-5931-82

	CD11b (Mac1) Ebioscience, clone M1/70, Cat. # 48-0112-82<br>CD45R/B220 BD Pharmingen, clone 30-F11 Cat. # 558108<br>CD3 Ebioscience, clone 145-2C11 Cat. # 48-0031-82<br>CD5 Ebioscience, clone 53-7.3, Cat. # 48-0051-82<br>CD117 (c-kit), Ebioscience, Clone 2B8, Cat.17-1171-82<br>Ly-6A/E (Sca), Ebioscience, Clone D7, Cat. # 25-5981-82<br>CD105 Ebioscience, Clone MJ7/18, Cat. # 25-1051-82<br>CD16/32 Ebioscience, Clone 93, Catt # 12-0161-83<br>CD34 Ebioscience, Clone RAM34, Cat. # 11-0341-85<br>CD150 Biolegend, Clone TC15-12F12.2, Cat. # 115912<br>CD48 Biolegend, Clone HM48-1, Cat. # 103432<br>TGFb1, Abcam, Clone EPR18163, Cat# ab179695<br>PF4, Abcam, EPR17279-1, Cat # ab182988<br>p-Tyrosine, Millipore, 4G10, Cat# 05-321<br>pY397 FAK Cell signaling, polyclonal, Cat# 3283<br>pY576/577 FAK Cell signaling, polyclonal, Cat # 3281<br>FAK, Cell signaling, polyclonal, Cat# 3285<br>b1 Tubulin, kindly provided by Dr. Joe Italiano, Brigham and Women's Hospital, Boston
Validation	- GPIbalpha, Emfret analytics, clone Xiao G5 and Xiao G7 were validated by Emfret analytics for flow cytometry use.<br>- Laminin: Specificity of the anti-laminin antibodies is determined by dot blot immunoassay. In the dot blot immunoassay the rabbit anti-laminin antibody reacts with laminin but not with fibronectin, vitronectin, collagen IV, or chondroitin sulfate types A, B, and C.<br>- beata1 integrin antibody, ab183666 was validated by Abcam for use by western blot<br>- beata1 integrin, becton Dickinson, clone Ha2/5, Cat# 555005 was validated by BD by flow cytometry. Was validated by our lab by using beta1 integrin KO cells.<br>- Activated beata1 integrin, Becton Dickinson, clone 9EG7, Cat# 553715 was validate by Lenter et al, PNAS, 1993.<br>-CD41, Ter119, Gr1, CD11b, B220, CD3, CD5, CD117, Sca, CD105, CD16/32, CD34, CD150, CD48, are routinely tested by flow cytometric analysis. by producers and literature.<br>- Anti TGFbeta1 and anti PF4 antibodies were tested by abcam for western blot use.<br>-Anti p-Tyrosine is routinely used in literature and it has been previously validated in our laboratory<br>- Anti FAK, pY397 FAK and pY576/577 were validated for western blot use by Cell Signaling and widely used in literature<br>-Anti beta1 tubuli antibody was validated by Dr. Joe Italiano's laboratory

Animals and other organisms

Policy information about studies involving animals; ARRIVE guidelines recommended for reporting animal research

Laboratory animals	Mouse 129S1/C57Bl/6, 6-12 week old, females and males were used<br>Mouse, NOD scid gamma (NSG), 8-12 week old, males
Wild animals	No wild animals were used
Field-collected samples	The study did not involve samples collected in the field
Ethics oversight	Mice were maintained and treated as approved by the Harvard Medical Area Standing Committee on Animals according to National Institutes of Health standards as set forth in the Guide for the Care and Use of Laboratory Animals

Note that full information on the approval of the study protocol must also be provided in the manuscript.

Flow Cytometry

Plots

Confirm that:

- [x] The axis labels state the marker and fluorochrome used (e.g. CD4-FITC).
- [x] The axis scales are clearly visible. Include numbers along axes only for bottom left plot of group (a 'group' is an analysis of identical markers).
- [x] All plots are contour plots with outliers or pseudocolor plots.
- [x] A numerical value for number of cells or percentage (with statistics) is provided.

Methodology

Sample preparation	Platelets: Blood was withdrawn by retroorbital bleed in Aster-Jandl anticoagulant, Blood was diluted 1:100 in PBS. 5 ul of diluted blood was stained with antibodies for 20 minutes in the dark. Samples were diluted with 300ul of PBS and acquired.<br><br>Bone marrow cells were flushed in 2% fetal bovine serum. Red blood cells were lysed annd samples were stained for 20 minutes

with specific antibodies. 400 ul of PBS was added and samples were analyzed within 1 hour.

Instrument

Becton Dickinson, FACS Calibur
Becton Dickinson, LSRII

Software

Becton Dickinson CELLQuest software
Becton Dickinson FACSDiva
FlowJo

Cell population abundance

Mouse studies:
Hematopoietic stem and progenitor cell populations were analyzed in samples of mouse total bone marrow cells.
Abundance of each population in control samples expressed as % of live bone marrow cells was:
Long term HSC: 0.02%
Short term HSC: 0.01%
Multipotent progenitors: 0.2%
CMP: 0.6%
GMP: 0.2%
PreMegE: 0.2%
MKP: 0.06%
Megakaryocytes (bone marrow): 1.5
Megakaryocytes (cultured): 80-90%
Ter119+ cells: 24%
CD3+ cells: 1.1%
B220+ cells: 30%
CD11b+cells: 47.8%

Gating strategy

hematopoietic stem and progenitor cells gating strategy:
1) Bone marrow cells were identified based on FSC/SSC.
2) Doublets were excluded based on FSC-H and FSC-A
3) Live bone marrow cells were selected by gating on DAPI negative events
4) Lineage negative cells were selected by gating on cells negative for lineage markers (Terr119, CD11b, CD3, CD5, Ly-6G (Gr-1) , CD45R/B220
5) LSK cells were gated as Lineage negative, Sca+, Kit+
6) LK cells were gated as Lineage negative, Sca-, Kit+
7) Long term HSC: Lin-, cKit+, Sca1+, CD48-, CD150+
8) Short term HSC: Lin-, cKit+, Sca1+, CD48-, CD150-
9) Multipotent progenitors: Lin-, cKit+, Sca1+, CD48+
10) PreMegE: cKit+, Sca1-, CD41-, FcgRI/IIlo, CD105+, CD150-
11) MKP: Lin-, cKit+, Sca1-, CD41+, CD150+
12) CMP: Lin-, cKit+, Sca1-, CD34+, FcgRI/II-
13) GMP: Lin-, cKit+, Sca1-, CD34+, FcgRI/II+
14) Megakaryocytes were identified based on their FSC and positivity for CD41 and CD42b

Controls for appropriate gating and compensation included unlabeled sample, single stained samples. For rare populations FMO control (Fluorescence Minus One Control) was used.

Platelets were identified based on their FSC/SSC and their positivity for CD41 or CD42b. Boundaries between positive and negative staining was defined using isotipic control antibodies

☒ Tick this box to confirm that a figure exemplifying the gating strategy is provided in the Supplementary Information.
